# Defining Differentially Methylated Regions Specific for the Acquisition of Pluripotency and Maintenance in Human Pluripotent Stem Cells via Microarray

**DOI:** 10.1371/journal.pone.0108350

**Published:** 2014-09-24

**Authors:** WenYin He, XiangJin Kang, HongZi Du, Bing Song, ZhenYu Lu, Yuling Huang, Ding Wang, Xiaofang Sun, Yang Yu, Yong Fan

**Affiliations:** 1 Key Laboratory for Major Obstetric Diseases of Guangdong Province, Key Laboratory of Reproduction and Genetics of Guangdong Higher Education Institutes, The Third Affiliated Hospital of Guangzhou Medical University, Guangzhou, China; 2 Center of Reproductive Medicine, Department of Obstetrics and Gynecology, Peking University Third Hospital, Beijing, China; 3 Union Stem Cell & Gene Engineering Co., Ltd., Tianjin, China; China Agricultural University, China

## Abstract

**Background:**

Epigenetic regulation is critical for the maintenance of human pluripotent stem cells. It has been shown that pluripotent stem cells, such as embryonic stem cells and induced pluripotent stem cells, appear to have a hypermethylated status compared with differentiated cells. However, the epigenetic differences in genes that maintain stemness and regulate reprogramming between embryonic stem cells and induced pluripotent stem cells remain unclear. Additionally, differential methylation patterns of induced pluripotent stem cells generated using diverse methods require further study.

**Methodology:**

Here, we determined the DNA methylation profiles of 10 human cell lines, including 2 ESC lines, 4 virally derived iPSC lines, 2 episomally derived iPSC lines, and the 2 parental cell lines from which the iPSCs were derived using Illumina's Infinium HumanMethylation450 BeadChip. The iPSCs exhibited a hypermethylation status similar to that of ESCs but with distinct differences from the parental cells. Genes with a common methylation pattern between iPSCs and ESCs were classified as critical factors for stemness, whereas differences between iPSCs and ESCs suggested that iPSCs partly retained the parental characteristics and gained de novo methylation aberrances during cellular reprogramming. No significant differences were identified between virally and episomally derived iPSCs. This study determined in detail the de novo differential methylation signatures of particular stem cell lines.

**Conclusions:**

This study describes the DNA methylation profiles of human iPSCs generated using both viral and episomal methods, the corresponding somatic cells, and hESCs. Series of ss-DMRs and ES-iPS-DMRs were defined with high resolution. Knowledge of this type of epigenetic information could be used as a signature for stemness and self-renewal and provides a potential method for selecting optimal pluripotent stem cells for human regenerative medicine.

## Introduction

DNA cytosine methylation is an important epigenetic modification in mammals that contributes to cell growth, differentiation, and particularly, early embryonic development [1], [2], [3]. Thus, DNA methylation profiles specifically reflect cell types and fates. Transformation of human induced pluripotent stem cells (iPSCs) from somatic cells requires a process of epigenetic reprogramming that is promoted by transient ectopic expression of defined transcription factors expressed in ESCs [4], [5], [6]. iPSCs share similar properties with human embryonic stem cells (hESCs), including the maintenance of the stem cell state and the potential for differentiation [7]. Sustained efforts have been made to identify the critical roles of DNA methylation in the induction and maintenance of pluripotency. Inhibiting the activity of DNMTs with 5-azacytidine (AzaC) or partially depleting DNMT1 promotes a fully reprogrammed state in somatic cells [8], implying a key role for methylation in the initial period of iPSC generation. iPSCs have been reported to acquire irregular methylation patterns during the reprogramming process while still possessing inherited DNA methylation states as epigenetic memories from parental cells [7], [9], [10], [11], [12], [13], [14], [15]. Moreover, aberrant epigenetic reprogramming has recently been reported in human iPSCs [7], [12]. The above reports suggest that methylation profile may represent an epigenetic signature, which was demonstrated to partially be a consequence of de novo methylation mediated by DNMT3B during reprogramming [16].

Compared with hESCs, iPSCs provide a valuable resource for regenerative therapies, particularly when immunematched, patient-specific pluripotent cells are needed. Retrovirus or lentivirus based delivery systems have been used as the mainstream methodologies for iPSC generation [17]. However, several recent studies determined that virally induced iPSCs harbor genetic and epigenetic aberrations that result in transcriptional abnormalities [18]. A diverse array of improved approaches has been used to generate non-integrative human iPSCs free of exogenous DNA. Episomal vectors, as non-integrative vectors, are appealing for their simple manipulation and high efficiency [17]. Additionally, episomal delivery is believed to be a step forward for stem cell therapy because of its low immunogenic potential compared with virally generated iPSCs [19]. Genetic stability and copy number variation have been compared between iPSCs generated using PiggyBac transposons and those created via retrovirus [20]. However, few studies have systematically investigated epigenetic differences among diverse iPSCs delivery strategies.

However, studies have reported the similarities and differences of various stem cell types in terms of genomic stability, transcriptomes [21], [22], [23], histone modifications [21], protein post-translational modifications [24] and DNA methylation [7], [10], [12], [13], [14], [25]. Genome-wide screens have been used to analyze epigenetic alterations in human pluripotent cells [26], [27]. In addition to experimental studies, comprehensive comparisons and meta-analyses performed by different laboratories have also increased the understanding of cellular DNA methylation. However, most of these previous studies were performed using Illumina's Infinium HumanMethylation27 BeadChip or reduced representation bisulfate sequencing, two methods that are suboptimal in terms of probe density and genome coverage. In our opinion, these methodologies are minimally informative for distinguishing unique cell signatures from one another because differentiated methylation profiles have been shown to be dramatically more complex than what had been previously thought. Thus, further validation of particular stem cell types using an advanced chip platform to precisely reveal details of DNA methylation profiles is required. This work may provide the following: first, a full view of genome methylation profiles of pluripotent cells; second, unique signatures that could distinguish pluripotent cell types from others; and third, a complete evaluation of the safety of pluripotent cells. In view of this, we compared the epigenetic DNA states of hESCs, hiPSCs reprogrammed using lentivirus and episomal vector, and their corresponding parental cells using the high-resolution Illumina Infinium HumanMethylation450 platform and identified a unique methylation pattern for each individual cell type. Our identified DNA methylation signatures provide a reference to improve the safety and flexibility of stem cell engineering applications.

## Materials and Methods

### Human cell culture and derivation of induced pluripotent stem cells

All experiments were approved by the ethical committee of The Third Affiliated Hospital of Guangzhou Medical University. The tissues donors provided their written informed consent for participation. Briefly, the donors were informed that their tissues were used for scientific research. Our research results will be published in a scientific research journal, but their names will not be emerged in the paper. They can not get any benefit in our research.

Human amniotic fluid (AF) was obtained by ultrasound-guided amniocentesis performed on pregnant women for routine prenatal diagnostic purposes at gestational ages ranging from the 18th to 22nd weeks. AF cells were obtained by the centrifugation of 10 ml of AF in a centrifuge tube at 1,000 rpm for 5 min. The supernatant was removed, and the cells were resuspended in 2 ml of AmnioMAX-II Complete Medium (Invitrogen, Carlsbad, CA, USA). The cells were then transferred to 6 cm dishes, and the volume was adjusted to 4 ml; these cells were cultured at 37°C under 5% humidified CO2. Cell clusters emerged at 7 days after seeding. Non-adherent cells were discarded. The cells were cultured and passaged routinely at 70–80% confluence. Fetal fibroblast (FF) cell lines were independently established in our laboratory from abortion fetal skin after obtaining informed consent. Human fibroblasts were cultured in fibroblast medium (Dulbecco's Modified Eagle's medium (DMEM) supplemented with 10% fetal bovine serum (FBS) (Hyclone, Logan, UT, USA), 1 mM glutamine (Gibco), l% non-essential amino acids (NEAA) (Gibco), and 100 IU/ml penicillin/streptomycin (Gibco)). Cells were infected with the STEMCCA lentiviral supernatants generated by the transfection of 293T packaging cells as previously described. To generate non-integrated iPS cells, AF cells were transfected with Episomal iPSC Reprogramming Vectors (Invitrogen, A14703) by electroporation, and the transfected cells were then plated onto vitronectin-coated culture dishes following the manufacturer's instructions.

Human iPSCs were established from AF and FF cells, which were designated AF-IPS-2, AF-IPS-3, AF-IPS-9, AF-IPS-11, FF-IPS-1 and FF-IPS-3. Human ES and iPS cell lines were cultured on Matrigel-coated tissue culture dishes (ES qualified; BD Biosciences) with mTeSR1 (STEMCELL Technologies, Vancouver, BC, Canada) at 37°C and 5% CO2 in a 100% humidified atmosphere incubator. The culture medium was refreshed daily until the cells were ready to passage or harvest. Cells were passaged by 1 mg/ml dispase (Gibco) every 3–4 days.

### DNA methylation analysis

DNA methylation analysis was performed using the Illumina Infinium assay with the HumanMethylation450 BeadChip (Illumina), and the BeadChip was scanned on a BeadArray Reader (Illumina) according to the manufacturer's instructions. Methylated and unmethylated signals were used to compute the ab-value, which was a quantitative score of DNA methylation levels that ranged from 0 for completely unmethylated to 1 for completely methylated DNA. On the HumanMethylation450 BeadChip, oligonucleotides for 485,577 CpG sites covering more than 14,000 genes are mounted, most of which are selected from promoter regions. CpG sites with a detection p value> 0.05 (computed from the background based on negative controls) were eliminated from the data during further analysis.

### Gene expression analysis

Total RNA was extracted from cells using TRIzol reagent (Invitrogen) according to the manufacturer's instructions. Complementary DNA (cDNA) synthesis was performed with the M-MLV Reverse Transcriptase Kit following the manufacturer's instructions. Quantitative RT-PCR was performed using a SYBR Greenbased PCR Master Mix (Takara) and signals were detected with ABI Stepone plus Real-Time PCR System (Applied BioSystems). The expression of Ube3a and Fgr were validated to see the consistency of methylation analysis (Ube3a-F: 5′-ACTGTGGCACTTTTCACCAT-3′, Ube3a-R: 5′-CTAAAGGCTGGCCCAGAAAA-3′, Fgr-F: 5′-AGCACCCCAGTTCTCCC-3′, Fgr-R: 5′-ATGATCCTTGGGAGGGGTC-3′).

### Web tools

The following web tools were used in this study: NIA Array (http://lgsun.grc.nia.nih.gov/ANOVA/) for hierarchical clustering, DAVID Bioinformatics Resources (http://david.abcc.ncifcrf.gov/tools.jsp), and Venn Diagrams (http://bioinfogp.cnb.csic.es/tools/venny/venny.php).

### Accession numbers

The Gene Expression Omnibus (GEO) accession number for the HumanMethylation450k BeadChip data which has been submitted to Gene Expression Omnibus in this paper is GSM1399275–GSM1399284.

## Results

### Establishment of human stem cell lines

Ten human cell lines, including 2 ESC lines, 6 iPSC lines and 2 parental cell lines, were used as a primary source for experimentation (Table 1). Human ESC lines hES-7 and hES-10 were derived in our laboratory from discarded embryos [28]. Human iPSC lines and AF-IPS and FF-IPS cell lines were independently established in our laboratory using infection with the STEMCCA lentivirus (containing doxycycline-inducible human OCT4, SOX2, KLF4 and c-MYC) or episomal vectors containing 6 factors (Oct4, Sox2, L-Myc, Lin28, Nanog and Klf4) from 2 fully differentiated parental cell types (AF and FF cells). These cells clearly showed human embryonic stem cell characteristics (Fig. S1).

**Table 1:** A list of human cells analyzed for a methylation state in this study.

Cell ID	Description	Ability of differentiation
AF	Human amniotic fluid cells(P3)	None
FF	Human fetal skin fibroblast cells(P5)	None
AF-IPS-2	AF lentivirus derived IPS cells (P10)	Pluripotent
AF-IPS-3	AF lentivirus vector derived IPS cells (P8)	Pluripotent
AF-IPS-9	AF Episomal vector derived IPS cells (P12)	Pluripotent
AF-IPS-11	AF Episomal vector derived IPS cells (P9)	Pluripotent
FF-IPS-1	FF lentivirus derived IPS cells (P15)	Pluripotent
FF-IPS-3	FF lentivirus derived IPS cells (P10)	Pluripotent

Numbers in parenthesis with P indicate passage in culture on the cells used in the methylation analysis.

### Analysis of genome-wide DNA methylation

**Figure 1:**
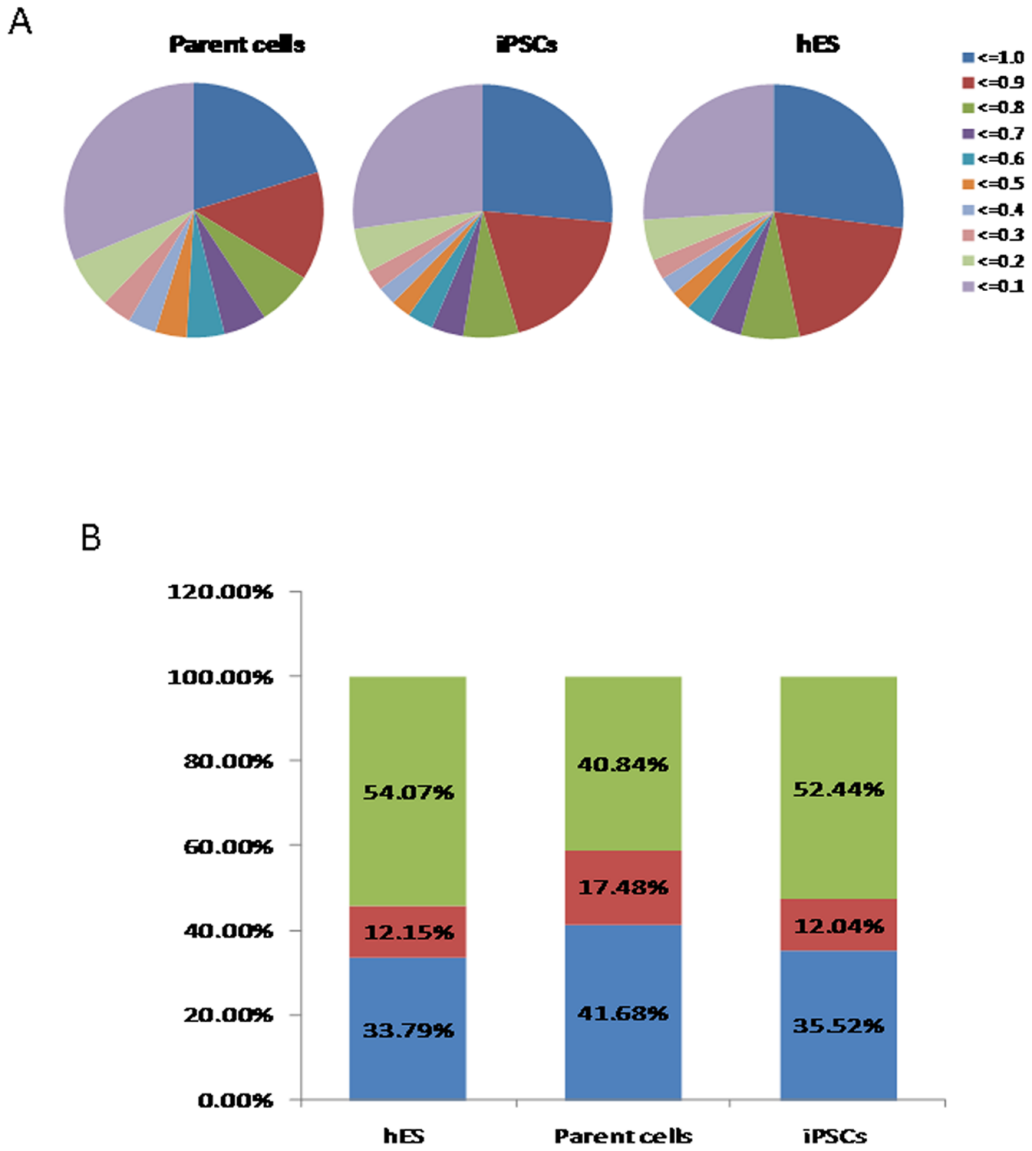
Pluripotent stem cells are significantly more hypermethylated than their corresponding parental cells. (A) Distribution of 485,577 CpG sites with their methylation scores in the parental cells, iPSCs and ESCs. (B) The average number of CpG sites with low (0–0.3), middle (0.3–0.7) and high (0.7–1.0) methylation. The iPSCs have more highly methylated sites than the parental cells.

**Figure 2:**
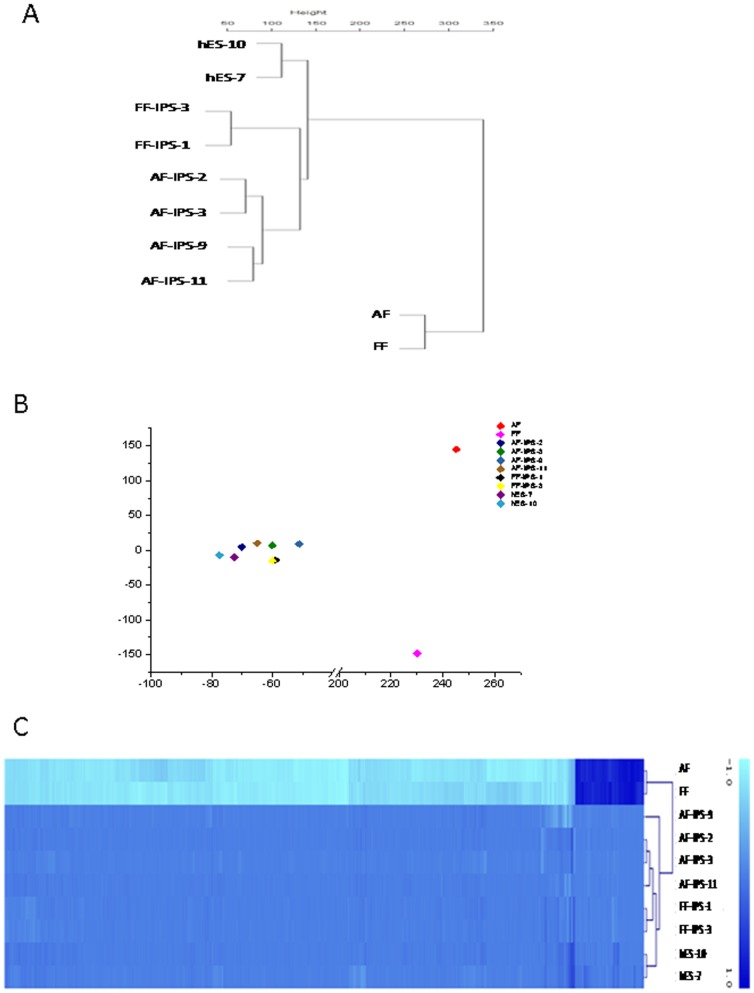
Pluripotent stem cells are significantly more hyper-methylated than differentiated cells. (A) Unsupervised hierarchical clustering analysis based on DNA methylation. (B) Principal component analysis (PCA) of the DNA methylation states of 485,577 CpG sites from 10 human cell lines. The principle component 1 axis clearly distinguishes the iPS/ES cell group from differentiated cells, whereas human iPS cells are very close to human ES cells. (C) Heat map showing hyper-methylation in human iPS/ES cells compared with differentiated cells. The heat map of hierarchical clustering analysis represents DNA methylation levels from completely methylated (dark blue) to unmethylated (light blue). Epigenetic distances (Euclidean Distance) were calculated by NIA Array.

To examine DNA methylation status in 8 human pluripotent stem cell lines and two differentiated cell lines (Table 1), we examined genome-wide DNA methylation using Illumina's Infinium HumanMethylation450 BeadChip, which interrogated over 480,000 CpG sites covering approximately 99% of RefSeq genes in the genome. DNA methylation levels in this assay system were recorded quantitatively using a scoring system ranging from 0 (completely unmethylated) to 1 (completely methylated). Using multiple repetitions, 485,577 methylated sites in 21,221 genes were analyzed from the 10 samples and categorized into three groups: Low (score≤0.3), Middle (0.3<score≤0.7), or High (score>0.7) methylation. The global distribution of DNA methylation levels is shown in Fig. 1A, with a similar profile observed among the pluripotent stem cells. The percentage of CpG sites in the High class in iPSC/ESC lines was 53.26% on average, whereas the percentage in differentiated cells was 40.84% (Fig. 1B). Meanwhile, approximately one-third of the examined CpG sites had a low level of methylation in both iPSCs and ESCs, whereas the percentage of sites with low methylation in differentiated cell groups reached 41.68% (Fig. 1B). These data imply that the methylation level of CpG sites was significantly higher in pluripotent stem cells than in differentiated cells. Scatter plot assays clearly distinguished iPSCs/ESCs from the differentiated cells (Fig. 2A) without showing significant variance among iPSC strains of the same origin (Fig. 2B). According to the heatmap of hierarchical clustering analysis, hypermethylated sites (shown in dark blue) were more widespread in iPS/ES cells compared with the differentiated cells (Fig. 2C), suggesting that gene promoters in iPSCs/ESCs were hypermethylated compared with those in differentiated cells.

### Identification of stem cell-specific differentially methylated regions (DMRs)

**Figure 3:**
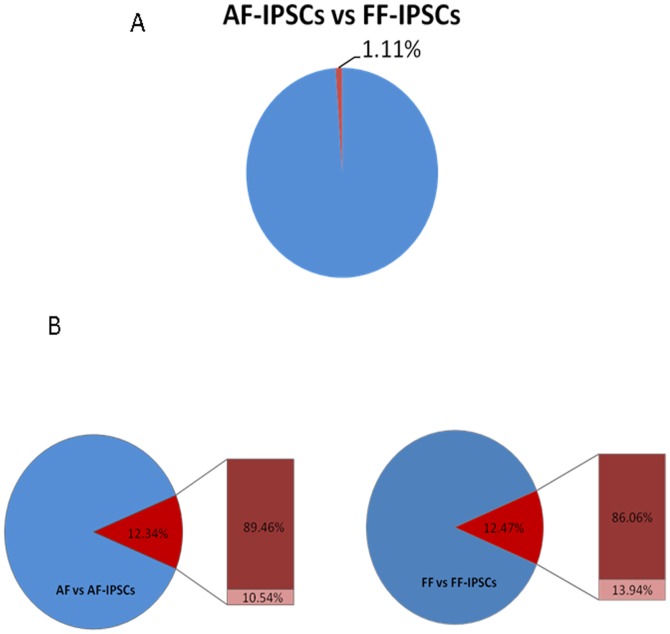
The ratio of CpG sites in iPSCs was significantly larger than that of the differentiated cells. (A) Comparisons of 485,577 CpG sites between two groups show high similarities between AF-IPS and FF-IPS cells. (B) In contrast, 12.34% and 12.47% of CpG sites are differentially methylated in AF-IPS and FF-IPS cells, respectively, compared with their parental cells (AF and FF). It should be noted that 89.46% and 86.06% of the differentially methylated sites (DMSs) are hypermethylated in AF-IPS and FF-IPS cells, respectively, compared with their parental cells.

**Figure 4:**
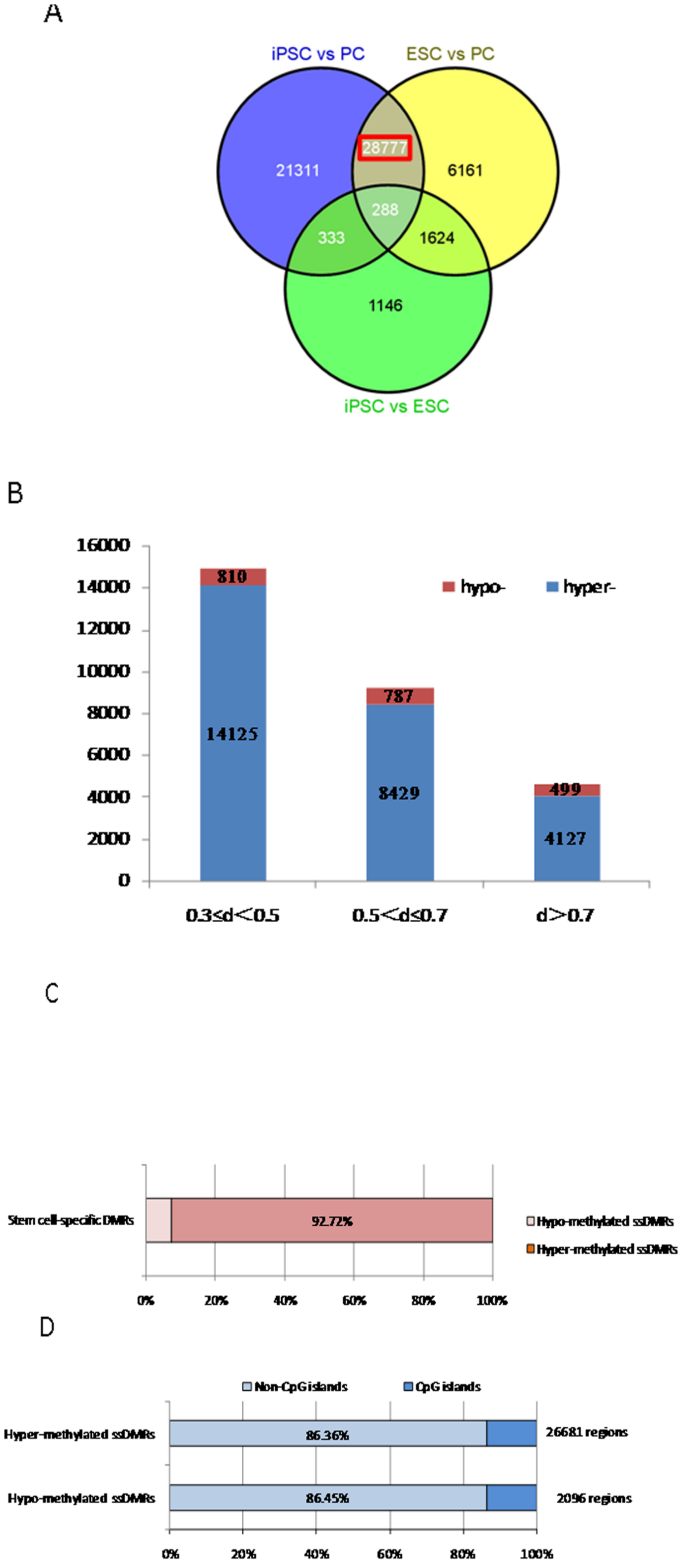
Venn-like diagram showing stem cell-specific differentially methylated regions (SS-DMRs) that overlap CpG sites among ESCs, iPSCs and their parental cells. (A) A total of 28,777 ssDMRs were identified. (B) The number of sites with low, middle and high methylation status. (C) In the pluripotent stem cells, 92.72% of the SS-DMRs are hypermethylated. (D) A total of 86.36% of the hypermethylated SS-DMRs and 86.45% of the hypomethylated SS-DMRs are located outside of CpG islands.

To focus on the specific changes in methylation levels observed between stem cells and differentiated cells, differentially methylated sites (DMSs) representing CpG sites with p-values differing by 0.3 points or more between the two cell groups were defined and analyzed. The DMSs between AF-IPS and FF-IPS cells were only 1.11% of all of the CpG sites (Fig. 3A), suggesting that iPSCs derived from different somatic cells had similar methylation states. Nevertheless, the DMSs between AF and AF-IPS cells and between FF and FF-IPS cells increased to 12.34% and 12.47%, respectively, implying that the methylation status of iPS cells was significantly different from that of their parental cells (Fig. 3B). It was also noted that among the DMSs between the iPS cells and their parental cells, approximately 90% changed from a hypomethylated state to a hypermethylated state in iPSCs. Furthermore, comparisons among ESCs (averaged from 2 lines), iPSCs (averaged from 6 lines) and parental cells (averaged from 2 lines) showed that 28,777 sites were detected as stem-specific DMRs (ssDMRs), whereas the remaining approximately 94.07% of the examined CpG sites did not show differential methylation among the strains (Fig. 4A). This result suggested that only a small number of the CpG sites were affected during the gain and maintenance of pluripotency. The 26,681 sites (92.72%) of the stem cell-specific DMRs had significantly higher methylation levels in iPSCs/ESCs than in the parental cells (Fig. 4B). In contrast, 2096 sites (7.28%) of the stem cell-specific DMRs were hypo-methylated in iPSCs/ESCs compared with the parental cells (Fig. 4C). Gene ontology analysis indicated that the hypomethylated stem cell-specific DMRs included genes related to the regulation of mRNA transcription and embryonic development, which mainly clustered into the Wnt, MAPK, Hedgehog, and TGF-β signaling pathways and other pathways involved in cancer (Fig. S2A, S2B). Interestingly, the majority of both the hypomethylated (86.45%) and hypermethylated (86.36%) stem cell-specific DMRs were located in non-CpG islands (Fig. 4D), which contrasts with observations in a previous study [11].

### Effect of reprogramming on DNA methylation status in iPSCs

**Figure 5:**
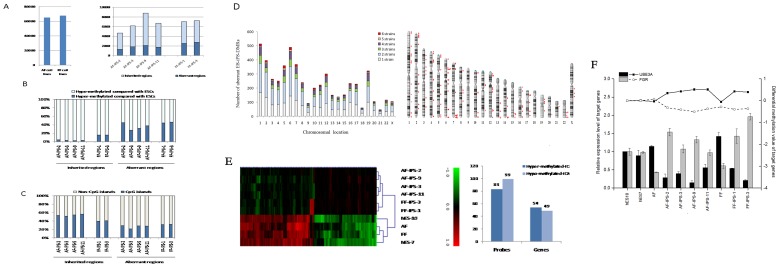
Comparison of aberrant and inherited methylation in human iPSCs. The DMRs between ESCs and iPSCs are designated as ES-iPS-DMRs, and the DMRs between ESCs and parental cells are designated as ES-parental-DMRs. (A) A comparison of ES-iPS-DMRs from iPSC lines derived from each parental cell are shown (left). The number of regions inherited from parental cells in iPSCs (light blue) and the aberrant regions in iPSCs that differ from ESCs and parental cells (dark blue) in the ES-iPS-DMRs are shown as bars (right). (B) The proportion of the hyper- and hypo-methylated ES-iPS-DMRs in inherited and aberrant regions of each iPSC line. (C) The proportion of the ES-iPS-DMRs associated with CpG islands and non-CpG islands in inherited and aberrant regions of each iPSC line. (D) The number and distrubition of overlapping aberrant ES-iPS-DMRs in iPSCs. Chromosome ideogram showing the location of the individual DMRs specific for iPSCs from this study. Red dots indicate the location of the individual DMRs covering a particular gene; red circles denote those for intergene region. (E) Clustering analysis of aberrant ES-iPS-DMRs. The heat map shows the methylation pattern of aberrant DMRs in human iPS cells compared with ES cells. The heat map of hierarchical clustering analysis represents DNA methylation levels from completely methylated (red) to unmethylated (green). Epigenetic distances (Euclidean Distance) were calculated by NIA Array. The number of probes and the genes representing hyper and hypomethylation are shown (right). (F) Gene expression level of Ube3a and Fgr. Bar and line graphs showing the normalized expression level and the DNA methylationb-values for two interesting genes. Gene expression was normalized to the expression of Gapdh. The mean values were calculated from independent experiments performed in triplicate.

To address the effect of reprogramming on the DNA methylation profile of human iPSCs, we compared the DNA methylation states of each iPSC line or each parental cell line with that of ESCs (averaged value) as a control (Fig. 5A). Genome wide, the number of DMRs between ESCs and iPSCs, termed ES-iPS-DMRs, varied among the 6 iPSC lines. Similarly, the number of DMRs between ESCs and the parental cells (ES-PC-DMRs) was also identified in the 2 lines. According to the comparison between ES-iPS-DMRs and ES-PC-DMRs, 3375 to 6753 inherited methylation sites from the parental cells were detected in individual AF-IPSCs, whereas the number in FF-IPSCs was approximately 4550 on average (Fig. 5A). In addition to methylation sites that were inherited from the parental cells, ES-iPS-DMRs were composed of aberrant methylation sites, which were iPS specific and more consistent in number among different strains than the inherited sites. Of the inherited regions, approximately 3.8% in AF-IPSCs and 16.0% in FF-IPSCs had a high level of methylation; this percentage was significantly lower than that of the hypomethylated sites (Fig. 5B), implying a widespread decrease in the expression of the majority of genes caused by reprogramming of parental cells. However, approximately 35.4% of aberrant ES-iPS-DMRs were hyper-methylated, which was slightly lower than the percentage of hypomethylated sites. Of the inherited sited in iPSCs, approximately 49.25% were located in CpG islands, whereas 50.75% were not. Meanwhile, a higher percentage (71.56%) of aberrantly methylated sites was located in non-CpG islands even in different iPSC lines (Fig. 5C). Thus, promoter regions in non-CpG islands were more affected during the reprogramming of the parental cells into pluripotent stem cells. The above results also indicated that despite different somatic cell origin and reprogramming strategy, iPSCs carried similar levels of aberrant methylation. Inspection of the recurrent aberrant DMRs in every iPSCs revealed that 52.3% (1905 out of 3643) of overlapped DMRs were located in chromosomes 1,2,5,6,7,12 and 19 (Fig. 5D). 182 of 3643 overlapped DMRs were shared among all 6 cell lines, suggesting regions resistant to reprogramming by either approach or origins. These iPSC-specific DMRs were distributed on each autosomal chromosome except chromosome 9 and 18. Notably, the subtelomeric region of some autosomal chromosome, as well as X-chromosome inactivation centre harboured a number of methylation variants. 65 out of these 182 sites were located in the intergene regions while the remains covered 103 genes. Clustering analysis indicated that approximately half of the ES-iPS-DMRs were hypermethylated in iPSCs compared with the parental cells and ES cells; these regions included the genes ZIC3, UBE3A and PDK1. The remaining ES-iPS-DMRs were specifically methylated at a low level in iPSCs. qPCR was performed to confirm the relative expression level of particularly interesting genes (Fig. 5F). UBE3A, an aberrantly hyper-methylated gene in iPSCs, was highly expressed in parental cells. Meanwhile, the expression of FGR, which was included in the aberrantly hypomethylated group, was up-regulated in iPSCs compared with other lines. Results of the gene expression and DNA methylation were consistent, implying a potential usage of these identified aberrant DMRs as molecular signatures of iPSCs.

### Identification of specific differentially methylated regions of virally and episomally derived iPSCs

**Figure 6:**
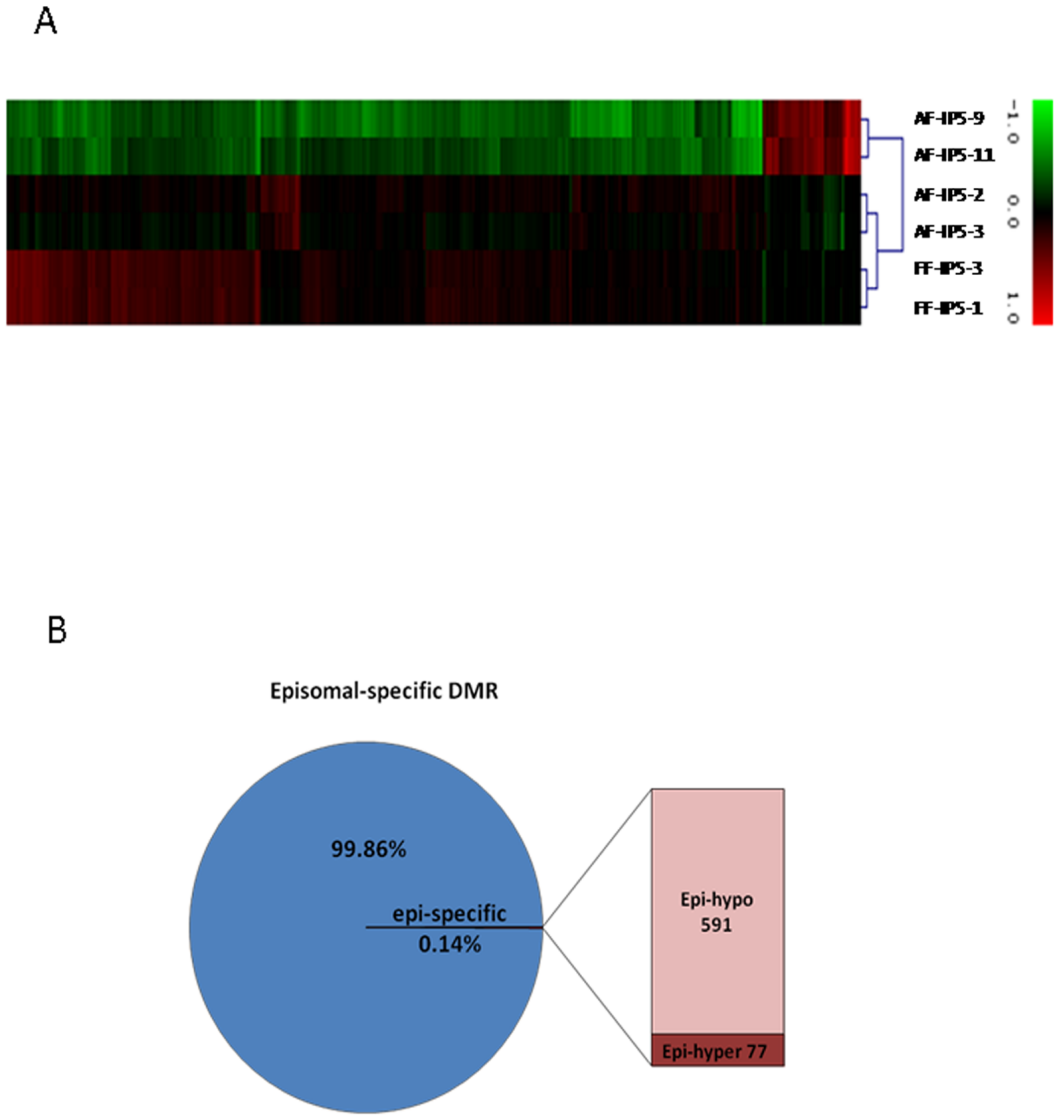
Clustering analysis showing differential methylation in episomal iPS cells compared with virally induced iPS cells. (A) The heat map of hierarchical clustering analysis represents DNA methylation levels from completely methylated (red) to unmethylated (green). (B) 0.14% of CpG sites are differential in episomally derived AF-IPS cells compared with virally derived AF-IPS cells. It should be noted that 591 of the DMRs are hypomethylated.

In general, independent of their method of generation, iPSCs of the same parental cells appeared in a tight cluster (Fig. 2A). However, to see the effect of different derivation methods on methylation, episomally derived AF-IPS-9 and AF-IPS-11 cells were grouped to compare their methylation states with those of the other four virally derived iPSCs. The results showed that only 0.14% of the total 485,577 sites were differentially methylated between cells derived with different methods (d>0.3, p<0.05), whereas 99.86% of detected sites appeared similar. Of these DMRs, 591 sites were hypermethylated in virally derived iPSCs; genes located in these regions are involved in viral myocarditis, antigen processing and presentation, ECM-receptor interactions, hematopoietic cell lineage and type I diabetes mellitus (Fig. 6). A total of 77 sites representing 20 genes were highly methylated specifically in episomally derived iPSCs and included OR4K14, PCDHGA2, PCDHGA3, PCDHGA1, KLHL4, FLJ26850, TMEM132D, C19orf41, FAM133A, NAP1L3, KCNC3, ZNF473, FZD10, CCDC85A, TMEM132C, NAPSB, OR4N5, C11orf80, PABPC5 and MYH14.

## Discussion

Over the past several decades, an increasing number of reports have individually focused on the distinct transcriptomes and methylomes of somatic cells, iPS cells, ES cells and uni-parental diploid cells [11], [14], [29], [30]. Several studies suggested large variations in iPSC-specific gene expression among separate data sets, at least on the level of the expression of individual genes [21], [22], [31]. Additional discrepancies existed in the formats of methylomic studies in the literature [12], [14]. There was limited overlap between the methylation signature identified by Huang's group and the signature genes identified in their former studies, and this signature was significantly different from what was previously described using either high-throughput sequencing or microarray analysis [16]. RRBS, which covered 10% of human CpG sites and was biased toward regions of high CpG density, was used in several previous studies to confirm the reported signatures in the literature, but poor reproducibility was found [7], [32], implying that this method is incapable of fully detecting true, consistent variances in iPSCs. BSPP covering 1% of all human CpG sites was recently used to identify signature genes that distinguish ESCs from iPSCs, although this technique still produced minimal overlap with the Infinium 27k array [25]. Given the lack of sample size, insufficient coverage was still considered the main challenge for the robust delineation of an accurate signature. The Infinium HumanMethylation450 BeadChip (Illumina), a newly developed chip containing over 450,000 methylation sites with over 99% of RefSeq genes covered and 98% above reproducibility, was chosen for this study to validate the methylation states of ESCs, iPSCs of different origins and generated by different protocols, and the parental somatic cells. These cells were comprehensively compared using this advanced platform, and the common properties and particular signatures of these cell types were precisely characterized under the same conditions.

Because iPSCs were derived from somatic cells and maintained “stemness” after reprogramming, this was an ideal model for studying the important role of methylation in regulating cell fate. Consistent with previous work, our genome-wide DNA methylation analysis determined that both iPSCs and ESCs existed in a general state of hypermethylation compared with differentiated cells (Fig. 1). Similar methylation profiles were observed among the pluripotent stem cells, whereas obvious differences were observed among somatic cells (Fig. 2). The identification of novel epigenetic markers may be an important tool for the validation of iPSCs and ESCs. In the present study, 28,777 ssDMRs were identified that were involved in the stem cell state, an amount that was increased compared with previous studies. The majority (92.72%) of these ssDMRs were hypermethylated in cells, implying a significant increase in genomewide methylation in pluripotent cells compared with somatic cells. This result also indicated that the reprogramming factors used during reprogramming activated only minimal numbers of stem-related genes by demethylation in parallel with methylating most genes that are associated with tissue-specific function. Furthermore, the 28,777 ssDMRs represented 8427 hypermethylated genes and 504 hypomethylated genes. Recently, eight genes, including SALL4, EPHA1, PTPN6, RAB25, GBP4, LYST, SP100 and UBE1L, were shown to be epigenetic markers for pluripotent stem cells [15]. Accordingly, in our study, EPHA1, GBP4, LYST and SP100 were exclusively found in the hypermethylated group, whereas SALL4 and RAB25 were hypomethylated. In particular, when combined with findings in the literature, GBP4 and SP100 were confirmed to be valid epigenetic markers for pluripotency [11], [15]. Gene ontology analysis showed that the genes associated with the hypomethylated ss-DMRs included a large number of transcription factors and proteins involved in embryonic development (Fig. 5). Some of these hypo-methylated genes might play a role in cellular dedifferentiation by becoming demethylated during global reprogramming, whereas others are correspondingly primed for activation upon differentiation. In contrast, most genes with SS-hyper-DMRs were found to play roles in differentiation pathways and functioned as part of particular processes, for example, metabolism or immune response, in differentiated cells, suggesting tissue-specific reactions were inhibited in stem cells.

It has been confirmed that the DNA methylation age of iPSCs is significantly younger than that of corresponding primary cells, with no significant difference detected between ESCs and iPSCs [33]. However, although iPSCs became very similar to ESCs after reprogramming, the number of aberrantly methylated sites was retained in iPSCs, suggesting that these two types of stem cells are not identical. The major difference was the increased instability of iPSC pluripotency, which was generally considered to be caused by “partial reprogramming” [9]. When compared with hESCs, iPSCs had specific required DMRs, termed ES-iPS-DMRs that were validated as a reflection of the memory influence of the parental cells and the efficiency of reprogramming in the present study. ES-iPS-DMRs were consistent with inherited regions, the sites that were remnants of the epigenomes of the donor cell, and aberrant regions that were uniquely identified in iPSCs but not in ESCs. Different somatic cells are of distinct genetic and epigenetic backgrounds, and iPSCs can be obtained at any timepoint and from various cell types. In this study, extraembryonic amniotic cells, characterized as having a normal karyotype, were expected to have a younger methylation state than FF cells. According to the results, the numbers of inherited regions varied among individual iPSCs even though the cells were from the same parental cell, reprogrammed using the same methods and cultured in the same conditions. Of these inherited regions, 1002 sites representing 363 genes were identified in both cell lines, whereas 1607 and 3024 sites were unique to a particular AF-IPS and FF-IPS cell line, respectively, and some corresponded to a particular iPSC clone. The existence of inherited regions has been termed epigenomic memory; it affected only a small percentage of genes and mainly caused a shift in the differentiation spectrum [10], [34], [35], [36]. We previously described the genomic stability of iPSCs and concluded that some CNVs and SNPs were introduced by reprogramming (data not shown), which is consistent with other studies [20], [37]. Thus, we speculated that the clone-specific DMRs were occasionally acquired as a result of genetic aberrance. Aberrant changes in DNA methylation between iPSCs and ESCs arising during reprogramming are a major concern. It has been proved recently that iPSC was not an improved pluripotent model as somatic cell nuclear transfer ES cell since the process occurs passively during factor-based reprogramming [38]. More effective reprogramming by SCNT was that the ooplasm provides ‘physiologic’ levels of reprogramming factors that were upstream of pluripotency. In contrast, transcription-factor-based reprogramming is associated with incomplete epigenetic reprogramming. The majority of methylation abnormalities in iPS cells were suggested resulting from reprogramming errors. In this study, there were a markedly higher number of aberrant regions in two FF-IPS strains than in AF-IPS strains, implying a greater induction of de novo methylation by reprogramming in this type of cell (Fig. 5A). In view of AF-IPS strains, it seemed that the aberrantly methylated variations for both virally- and episomal- reprogramming approaches were not statistically different, but this conclusion is limited by the small numbers of cell lines analysed. A list of 103 common genes carrying aberrant sites among all six iPSCs was further analyzed and showed an opposite methylation pattern to that of both ESCs and somatic cells. It has been widely suspected that de novo methylation plays an important role in establishing a unique iPSC methylation signature [16]. Furthermore, most of the identified ES-iPS-DMRs were hypomethylated, particularly those that were inherited (Fig. 5B). Although iPSCs, like ESCs, were reported to have a general hypermethylated status, it was clear that the genes containing hypomethylated iPSC-specific DMRs were required to be highly expressed under the driving force of reprogramming and played an important role during self-renewal [15]. Gene ontology analysis showed that the genes containing aberrantly hypomethylated sites clustered according to the major keywords of GTPase regulator and development.

Notably, we found that hypomethylated ssDMRs were abundant in non-CpG islands (Fig. 4), which contrasted with what Nishino's group described [15]. We found that not only hypo- but also hyper-methylated ssDMRs were biased for non-CpG island locations. Meanwhile, we determined that a majority of aberrant ES-iPS-DMRs preferentially occurred in non-CpG islands, which coordinate to previous studies [12]. Together, these results suggested that promoter regions in non-CpG islands are more directly affected by the gain and maintenance of pluripotency, indicating that iPSC reprogramming was less faithfully capable of resetting the DNA methylation and corresponding gene expression program. Our finding is supported by other related investigations: first, DNA methylation was revealed to primarily occur in non-CpG island regions of promoters in mouse ES cells [39], second, reprogramming of somatic cells into iPSCs was accompanied by extensive DNA methylation in CpG-poor regions with few CpG-rich promoters [40], and third, a recent whole-genome bisulphite sequencing was consistently showing that iPS cells carried threefold more aberrant CG and tenfold more aberrant non-CG methylation compared to NT ES cells.

To summarize, our experiments yielded highly reproducible results and deep coverage reads, which revealed that few hypomethylated genes initially participated in cellular self-renewal to co-regulate the acquisition of pluripotency, whereas a large number of hypomethylated genes played critical roles in maintaining the stem cells in a pluripotent state. The identification of ssDMRs as CpG methylation signatures in respective stem cell strains may help to artificially modify cells according to experimental and clinical requirements. In addition, knowledge about the precise DNA methylation profile in stem cells may enable a screening/evaluating of optimal stem cells for future human therapeutic applications.

## Supporting Information

Figure S1 **Immunohistochemistry of the stem cell-specific surface antigens OCT4, NANOG and TRA-1-60 in AF-iPSCs and FF-iPSCs and teratoma formation of those iPSCs by subcutaneous implantation into NOD/SCID mice.** The iPSCs differentiated into various tissues, including ectoderm (neural tissues), mesoderm (cartilage) and endoderm (glandular tissues).
(TIF)

Figure S2 **Annotation of ssDMRs.** (A) Annotation enrichment analysis of hypo-methylated ssDMRs. (B) KEGG pathway analysis of hypo-methylated ssDMRs. (C) KEGG pathway analysis of hyper-methylated ssDMRs.
(TIF)
