# Supplementary figures and images for: Defining Differentially Methylated Regions Specific for the Acquisition of Pluripotency and Maintenance in Human Pluripotent Stem Cells via Microarray
Source: PLoS One. 2014 Sep 24;9(9):e108350. doi: 10.1371/journal.pone.0108350 (PMC4177110; doi:10.1371/journal.pone.0108350)

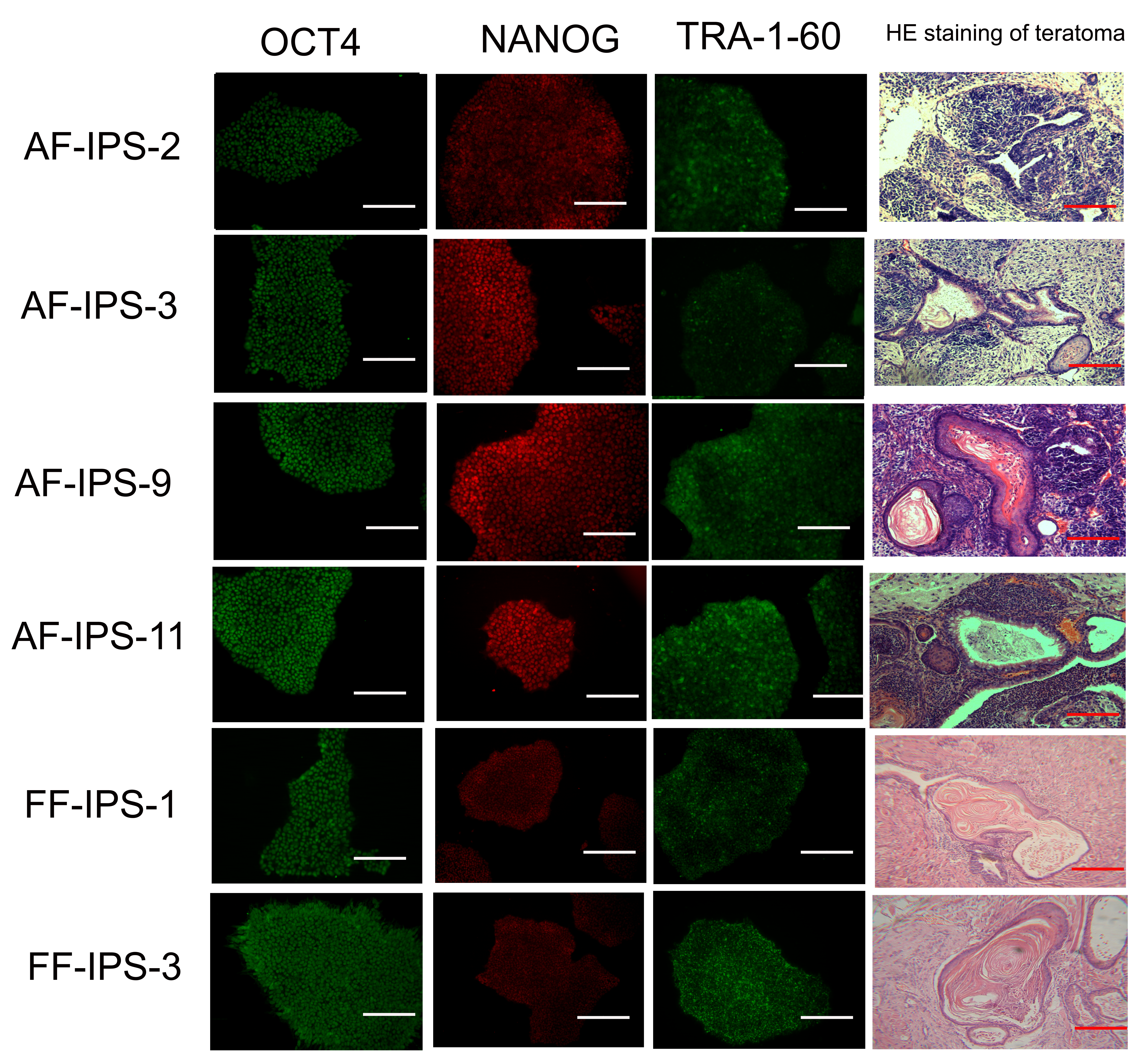

Supplement: Figure S1 — Immunohistochemistry of the stem cell-specific surface antigens OCT4, NANOG and TRA-1-60 in AF-iPSCs and FF-iPSCs and teratoma formation of those iPSCs by subcutaneous implantation into NOD/SCID mice. The iPSCs differentiated into various tissues, including ectoderm (neural tissues), mesoderm (cartilage) and endoderm (glandular tissues). (TIF) [file pone.0108350.s001.tif]

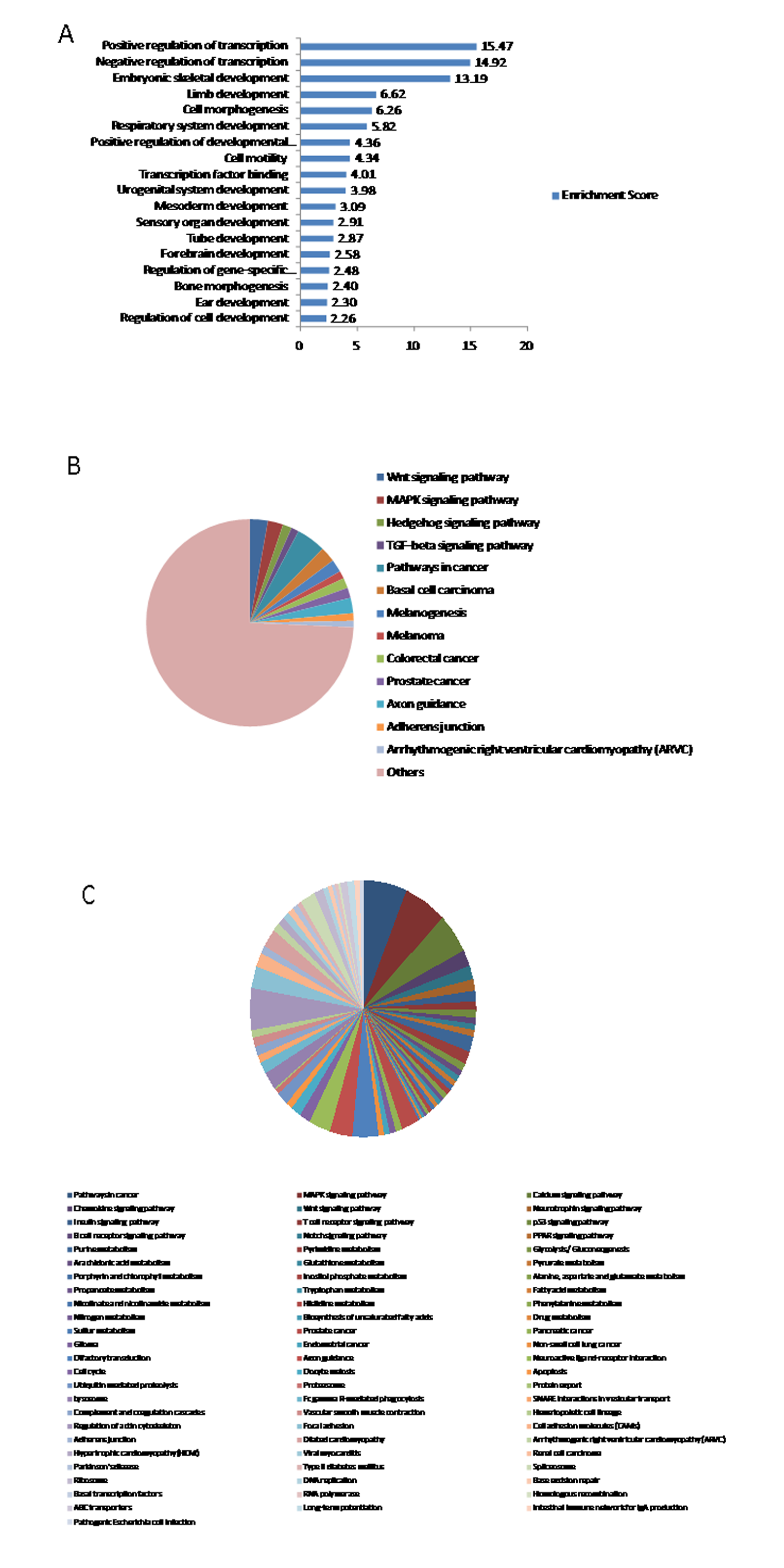

Supplement: Figure S2 — Annotation of ssDMRs. (A) Annotation enrichment analysis of hypo-methylated ssDMRs. (B) KEGG pathway analysis of hypo-methylated ssDMRs. (C) KEGG pathway analysis of hyper-methylated ssDMRs. (TIF) [file pone.0108350.s002.tif]
